# Opiate Drug Use and the Pathophysiology of NeuroAIDS

**DOI:** 10.2174/157016212802138779

**Published:** 2012-07

**Authors:** Kurt F Hauser, Sylvia Fitting, Seth M Dever, Elizabeth M Podhaizer, Pamela E Knapp

**Affiliations:** 1 Department of Pharmacology and Toxicology, Virginia Commonwealth University School of Medicine, Richmond, VA 23298, USA;; 2 Department of Anatomy and Neurobiology, Virginia Commonwealth University School of Medicine, VA 23298, USA;; 3 Institute for Drug and Alcohol Studies, Virginia Commonwealth University, Richmond, VA 23298, USA

**Keywords:** HIV, SIV, opiate drug abuse, NeuroAIDS, µ-opioid receptors, HIV-associated neurocognitive disorders (HAND), CNS, neurotoxicity, microglia, astroglia, oligodendroglia, myelin, neuroimmunology.

## Abstract

Opiate abuse and HIV-1 have been described as interrelated epidemics, and even in the advent of combined anti-retroviral therapy, the additional abuse of opiates appears to result in greater neurologic and cognitive deficits. The central nervous system (CNS) is particularly vulnerable to interactive opiate-HIV-1 effects, in part because of the unique responses of microglia and astroglia. Although neurons are principally responsible for behavior and cognition, HIV-1 infection and replication in the brain is largely limited to microglia, while astroglia and perhaps glial progenitors can be latently infected. Thus, neuronal dysfunction and injury result from cellular and viral toxins originating from HIV-1 infected/exposed glia. Importantly, subsets of glial cells including oligodendrocytes, as well as neurons, express µ-opioid receptors and therefore can be direct targets for heroin and morphine (the major metabolite of heroin in the CNS), which preferentially activate µ-opioid receptors. This review highlights findings that neuroAIDS is a glially driven disease, and that opiate abuse may act at multiple glial-cell types to further compromise neuron function and survival. The ongoing, reactive cross-talk between opiate drug and HIV-1 co-exposed microglia and astroglia appears to exacerbate critical proinflammatory and excitotoxic events leading to neuron dysfunction, injury, and potentially death. Opiates enhance synaptodendritic damage and a loss of synaptic connectivity, which is viewed as the substrate of cognitive deficits. We especially emphasize that opioid signaling and interactions with HIV-1 are contextual, differing among cell types, and even within subsets of the same cell type. For example, astroglia even within a single brain region are heterogeneous in their expression of µ-, δ-, and κ-opioid receptors, as well as CXCR4 and CCR5, and Toll-like receptors. Thus, defining the distinct targets engaged by opiates in each cell type, and among brain regions, is critical to an understanding of how opiate abuse exacerbates neuroAIDS.

## INTRODUCTION

There are compelling reasons to investigate opioid and HIV-1 interactions and their role in accelerated neuropathogenesis. Studies in HIV-1-infected opiate abusers show severe neuropathology compared to infected non-drug users [1-4], with selective increases in microgliosis [2, 4, 5]. In this cohort, multinucleated giant cells and HIV-1 p24 immunoreactive glia were found over 3-fold more frequently in injection drug users than in non-drug using HIV-1-infected individuals [1]. These initial findings were supported by subsequent histopathological confirmation [2, 3] indicating that opioid use can exacerbate the central nervous system (CNS) complications of HIV-1 infection. Despite some initial controversies discussed below, current studies now describe diminished cognitive function in HIV-1-infected individuals who preferentially abuse opiates even in patients who are receiving combined antiretroviral therapy (cART) therapy [6-8]. Prolonged heroin use exaggerates deficits in recall and working memory beyond impairments seen with HIV-associated neurocognitive disorders (HAND) (for definitions see [9-11]) alone [8]. Earlier controversies questioning the extent that opiate drug use worsens simian immunodeficiency virus (SIV)/simian-human chimeric immunodeficiency virus (SHIV) infection and neuropathogenesis may partially relate to the fact that some of the SIV/SHIV strains employed were not especially neurotrophic and that many of the studies failed to examine CNS pathology beyond CSF viral loads. An authoritative review covering opioid-SIV interactions appeared recently [12].

In the present review, the term “opiate” is used to refer to alkaloids derived from the opium poppy *Papaver somniferum* and includes opium and heroin [13]. Opiates act by mimicking endogenous “opioid” ligands and bind “opioid” receptors [14-20]. In the case of opiate drugs with abuse liability, they principally act by triggering the μ-opioid receptor (MOR) [13], and to a lesser extent δ- (DOR) and κ- (KOR) opioid receptors. Heroin is quickly deacetylated to morphine in the CNS and morphine is the main bioactive product of heroin in the brain [21, 22].

HIV-1 enters the brain early in the disease and establishes latent reservoirs in perivascular macrophages prior to the onset of HIV encephalitis (HIVE) [23]. Interestingly, preferential opiate abuse may selectively affect the turnover of the perivascular macrophage pool [4] and/or release cells chronically infected with HIV-1 from latency as assessed by increased LTR transactivation in a neuroblastoma cell line [24]. Opiates increase viral loads and hasten the progression and neuropathology in SIV models [25-29] (reviewed in [12]). Novel findings in SIV models also indicate that chronic opiate exposure can shape viral evolution [30-32].

Post-cART era studies find diminished cognitive function in HIV-1-infected individuals who preferentially abused heroin that is partially attributable to opioid abuse [6-8]. A recent study, examining the relationship of substance use history to neurocognitive impairment in HAND, indicated that “lifetime heroin dosage” correlated significantly with “poor recall and working memory” [8]. Since the participants in this study were currently abstaining from drug use, the findings indicate that heroin use can potentially result in lasting deficits to cognitive function in HIV-1-infected individuals [8]. A separate consideration is polysubstance abuse and the concept that opiate drug use interacts uniquely with other abuse substances to exacerbate HAND. For example, a rare fulminant encephalopathy with extensive basal ganglia involvement is associated with combined cocaine and heroin, i.e., “speedball”, use in a small subset of HIV-1-infected individuals [33]. Heroin, by virtue of immune suppression and increased HIV-1 replication, may worsen the inherent neurotoxic effects of cocaine and vice versa in neuroAIDS [33]. Nonetheless, some clinical studies have reported minimal or no neurocognitive differences between HIV-1-infected and uninfected drug abusers [34-36]. Clinical inconsistencies may be partially attributable to our lack of understanding of the mechanisms underlying drug and HIV-1 interactions and the spectrum of resultant comorbid manifestations [37-39]. Genetic risks for opiate abuse [40] or neuroAIDS, such as familial predisposition to dementia [41], MOR polymorphisms [42-44] and/or epigenetic changes in MOR [45], as well as polymorphisms in comorbid factors such as CCR5 [46-48], CCL2/MCP-1 [49], apolipoprotein ε (ApoE) allelic variations [50] (the ApoE4 allele has been linked to HIV-1 dementia and neuropathy [51, 52]) and prodynorphin [53-58] may also contribute to the complexity. In addition, street drug impurities [38], variable pharmacokinetics [59] and usage/dosage regimen [38], as well as the timing and duration of opiate exposure during the course of HIV-1 infection [39, 60], also are likely to determine the nature and severity of the comorbidity.

## THE CNS IS PREFERENTIALLY SUSCEPTIBLE TO OPIATE DRUG-HIV-1 INTERACTIONS

The heightened vulnerability to opiate drug actions in HIV-1-infected individuals [39, 61-65] results from a coordinated and exaggerated response of glia, and especially astroglia. Not only does morphine directly affect the response of neurons [66, 67] (including human neurons [50, 68, 69]), but opiates can directly affect MOR-expressing astroglia [70-79], microglia [61, 66, 80-82], oligodendroglia [83], and glial precursors [84, 85]. Morphine’s unique actions in HIV-1-exposed astroglia, in particular, appear to drive spiraling, intercellular feedback loops with microglia and perivascular macrophages that increase and sustain inflammation [39, 71]. In fact, unlike other HIV-1-infected "end organs" [target organs], which also contain subsets of MOR-expressing resident and newly recruited macrophages, the brain differs in the unique and exaggerated response of astroglia to opioids [39]. This is especially apparent in striatal astrocytes, which show far more pronounced morphine-HIV-1 protein-induced cytokine production [70, 72] compared to astrocytes isolated from the cerebral cortex, cerebellum, or spinal cord [86], and may contribute to the enhanced neuropathology seen in regions of the basal ganglia.

## NEUROTOXICITY WITH CHRONIC OPIATE EXPOSURE

With more sensitive and sophisticated approaches for assessing neuronal injury, emerging evidence suggests that sustained morphine exposure may be intrinsically neurotoxic. While there has been sporadic experimental evidence especially using cell culture models that opiates can be cytotoxic to neurons, as well as other cell types, only a few clinical studies reported modest astrogliosis in opiate abusers [87]. Rarely, severe astrogliosis is reported with heroin abuse [87-89] or in individuals inhaling volatilized heroin vapor [89, 90]. By contrast, in another study, dopaminergic function, as assessed by tyrosine hydroxylase (TH) terminals, was significantly reduced in the nucleus accumbens; while an index of serotonergic (5-hydroxyindoleacetic acid) function and TH was variably affected in the putamen and caudate nucleus [91]. Reports of increased perivascular infiltrates of lymphocytes and macrophages were also noted [92]. However, the nondescript nature of the gliosis in the above instances was potentially attributable to the physical and psychological "side effects" of addiction, including poor nutrition, poor health and lack/avoidance of medical care, and/or a marginal life-style [89, 90]. More recently, hyperphosphorylated tau was described in hippocampal neurons of a carefully characterized cohort of preferential opiate abusers [93]. In this cohort, the authors were careful to control for risk factors such as age and neurodegenerative or infectious diseases that could confound the interpretation of the deleterious effects of chronic opiate abuse *per se* [93]. Similar neuropathology characteristic of the aged brain involving tau hyperphosphorylation and increased amyloid and amyloid precursor disposition was seen in opiate abusers less than 40 years of age [94].

## METHODS TO MEASURE NEUROTOXICITY

Cell culture combined with computer-guided, time-lapse microscopy enables dramatic increases in throughput (see [95-97]) using well-established repeated measures designs to examine dynamic changes in the same neurons over time [98-104]. For example, this strategy reveals subtle, but significant, neurodegenerative effects after 60 h of sustained morphine exposure [95] that are even more evident after 72 h [60]. These otherwise subtle morphine-induced neuron losses and synaptodendritic injury (in preparation) were less apparent when examining average changes in populations of neurons. Depending on the outcome measure, the heterogeneity within the same class of neuron from a particular brain region [e.g., see  105] can preclude studying average changes in populations of neurons. This is particularly true for examining subcellular changes in the genesis and degeneration of individual synapses, which by necessity rely on repeated-measure design strategies [98-104]. This approach also enables meaningful analysis of small numbers of cells (e.g., rare samples of human neurons) or to discriminate among subsets of neurons (e.g., differentiating transfected versus non-transfected neurons in the same culture dish [106]).

The advantage of using repeated measures time-lapse microscopy is that individual neurons (within a treatment group) are compared to themselves prior to and during treatment (these designs are a “block randomized” or “repeated measure” design). This eliminates intersubject variability and differences each time new cell cultures are established, and is fundamentally more sensitive than examining cell populations. It is a common approach in electrophysiological studies and has been used to study neuron differentiation and death [e.g., see 101, 107]. The assay permits the use of repeated measures ANOVA, rather than regular multi-way ANOVA, and provides far greater statistical power with a small number of experiments [95, 97, 108, 109]. Moreover, although the strategy is used to assay neuronal cell death, it is equally valuable in structural and functional assessments of non-lethal synaptodendritic alterations in neurons and glia. It is important to note, in a repeated measure analysis, significance is not reflected in the error bars, which do not correspond to the error term used in the statistical analyses. Statistically, the response of an individual neuron can be correlated to itself over time, and the repeated measures design extracts the individual variation in the same neurons over time from the error term, increasing the power to detect treatment differences [110].

## NEUROAIDS IS GLIALLY-MEDIATED

HIV-1 infection in the brain is almost exclusively limited to microglia, latently infected astroglia [111], and astroglial progenitors [112]. Neuron injury or death is mainly through bystander effects as neurons, which lack the CD4 receptor, are not infected by the virus. Moreover, although endothelial cells and perivascular macrophages can become infected with HIV-1, intervening glia (especially astroglia) are typically present between infected endothelia or perivascular macrophages and neurons. By virtue of harboring HIV-1 or through aggressive attempts to control the infection, glia become the sources of toxins, which cause bystander effects on neurons. Thus, neuronal injury in HIV-1-infected individuals is caused by intercellular (glia-to-neuron) transfer of neurotoxic signals [38, 113-117].

## SUBLETHAL NEURON CHANGES UNDERLIE HIV-ASSOCIATED NEUROCOGNITIVE DISORDERS

The dendritic arborizations and synaptic connectivity of neurons are reduced in neuroAIDS [118] and the losses in connectivity are the likely substrates of behavioral and cognitive impairment in HAND [119-123]. Similarly, dendritic pruning and synaptic culling have been suggested as underlying comorbid opiate drug-HIV-1 interactive deficits in CNS function [2, 124]. Our recently published [124] and unpublished data suggest that synaptodendritic injury is highly correlated with (and likely underlies) the exaggerated neurobehavioral defects seen in opioid abuse-HIV-1 comorbidity. Although cumulative and sustained sublethal injury may ultimately result in reduced survival [124], the signaling events underlying opiate and HIV-1-induced sublethal injury may differ qualitatively from those regulating death, are potentially reversible, and more amenable to treatment and recovery.

If neuronal injury is associated with synaptodendritic injury, then why is neuron death typically described as an endpoint? Initial histopathological observations displayed evidence of neuronal and glial cell death at the end stages of neuroAIDS, especially in the pre-cART era. Early *in vitro* studies attempted to model the lethal changes. As noted, however, subsequent evidence increasingly supports the notion that neurobehavioral deficits are accompanied by sublethal synaptodendritic injury and a loss of neural circuitry [119-123]. While cumulative sublethal neuron injury may lead to neuronal death, ongoing studies from multiple laboratories clearly indicate that the extent of the damage is directly related to the concentration/dosage of HIV-1 proteins or HIV-1 titer. In fact, our published and unpublished findings indicate that synaptodendritic injury may be evident following exposure to concentrations of HIV-1 Tat that are 2-3 orders of magnitude less than required to induce rapid death of neurons (within 24-72 h) [60, 67, 95, 96, 106, 108, 109]. Lastly, even at high concentrations of HIV-1 proteins, sustained synaptodendritic injury precedes neuron death. We propose that as the concentrations of HIV-1 virions and HIV-1 Tat and gp120 proteins are reduced to levels normally seen in the HIV-1-infected CNS, the predominant neuronal alterations will be synaptic culling and pathophysiological changes in dendrites, without precipitous neuron death. Importantly, the sublethal changes are likely to be reversible and therefore highly amenable to therapeutic intervention.

## SYNAPTODENDRITIC ORIGINS OF EXCITOTOXIC INJURY, METABOLIC COMPROMISE, AND [Ca^2+^]_i_ OVERLOAD

HIV-1 gp120 and Tat overactivate glutamate receptors and are excitotoxic [125]. Tat [126-130] and gp120 [131-136] have been proposed to activate NMDA receptors (NMDARs) through direct and indirect mechanisms. In addition to the activation of NMDARs by Tat [126, 137], the virotoxin also interrupts mitochondrial function [138], ATP production [50, 139], and can cause focal, transient (“hit and run” [140]) disruptions to neuronal homeostasis through mechanisms responsible for dendrotoxicity and synaptic losses [140-142]. These may be compartmentalized to dendrites, axons, or perhaps specific synapses [143, 144]. Focal swelling [145] is accompanied by a disruption of ion homeostasis and ATP production [138, 146] causing a failure of neuritic transport [147-149]. Autophagosomes form at sites with collapsed cytoskeletal proteins and dendritic swelling [150], as do focal elevations in cleaved caspase-3 [151], respectively, suggesting roles for autophagy and “synaptic apoptosis” [152, 153]. Thus, Tat [154-156], gp120 [157, 158], and/or opiates [159] may trigger synaptodendritic injury through localized contributions from caspases, autophagic, and/or the ER-stress/unfolded protein response (UPR) effectors.

## OPIOID EXPOSURE IS LIKELY TO EXACERBATE THE EXCITOTOXIC EFFECTS OF HIV-1 AT THE LEVEL OF THE SYNAPSE

Endogenous opioids [95, 160, 161], as well as opiate drugs with abuse liability [162-167], have long-been known to reduce both the complexity of dendrites and the density of dendritic spines in a variety of brain regions. Interrelated studies describe how morphine can cull spines in pyramidal neurons from the cerebral cortex through a series of events involving NeuroD, microRNA-190, and Ca^2+^/calmodulin-dependent kinase II (CaMKII) [103, 168-170]. The ability of opiate drugs to reduce synaptic interconnections is highly selective. MOR-dependent reductions in dendritic spines display "agonist selective" responses. The "biased agonism" [102, 103] is reliant on the differential coupling of MOR to Gα, Gβγ, and/or β-arrestin by different MOR agonists. For example, morphine readily causes spine retractions, while fentanyl, a MOR agonist with much higher selectivity for MOR than morphine, fails to reduce spine numbers [170].

Morphine may potentially act through several MOR-dependent mechanisms to potentiate the excitotoxic effects of HIV-1; each will be explored below. Initially, morphine *via *Gβγ increases the activity of G-protein-gated inwardly rectifying K^+^ (GIRK/Kir3) channels making the neuron less excitable [171]. However, with chronic activation accompanied by tolerance (and dependence), MORs uncouple from the inwardly rectifying K^+^ channels [16], resulting in increased neuron excitability despite the sustained presence of opioid drugs. Morphine may also indirectly increase neuron excitability through actions in astroglia. Morphine can augment [Ca^2+^]_i_ *via *Gβγ [172, 173] (or perhaps uniquely *via *G_q/11_-α in astroglia [76]) to drive increases in phospholipase C (PLC), further increasing excitation. The resultant IP_3_-dependent increases in [Ca^2+^]_i_ potentiate Ca^2+^-induced Ca^2+^ release (or regenerative Ca^2+^) *via *ryanodine receptors in astroglia [76]. HIV-1 Tat can similarly increase [Ca^2+^]_i_ in astroglia [174] and restrict glutamate uptake [95], but is also a potent activator of NF-κB [70, 175] resulting in cytokine and chemokine release by astroglia [70-72, 176, 177]. In combination, morphine can potentiate Tat-induced increases in astroglial [Ca^2+^]_i _[72], reactive oxygen species (ROS) production [95], and IL-6, RANTES, and MCP-1 release [72], while causing subadditive restrictions in glutamate uptake [95]. Gp120 can also increase [Ca^2+^]_i_ [86, 178-180], alter cytokine expression [181], and limit glutamate uptake by astroglia [60, 179, 180, 182, 183], and likely interacts with opiate drugs through one or more of these mechanisms to increase neuron injury [60, 69]. By attenuating the presynaptic activity of inhibitory GABAergic interneurons, morphine can disinhibit postsynaptic target neurons thereby decreasing their excitotoxic threshold [184]. Morphine can cause significant increases in glutamate release by microglia [80], beyond that seen with Tat treatment alone [80]. Lastly, we speculate that the excessive astroglial response further exaggerates the intrinsic microglial response to HIV-1 [71], creating an opioid-driven, astroglial-to-microglial escalating feedback loop, which increases neuron injury [39, 61, 63, 80].

**Fig. (1):**
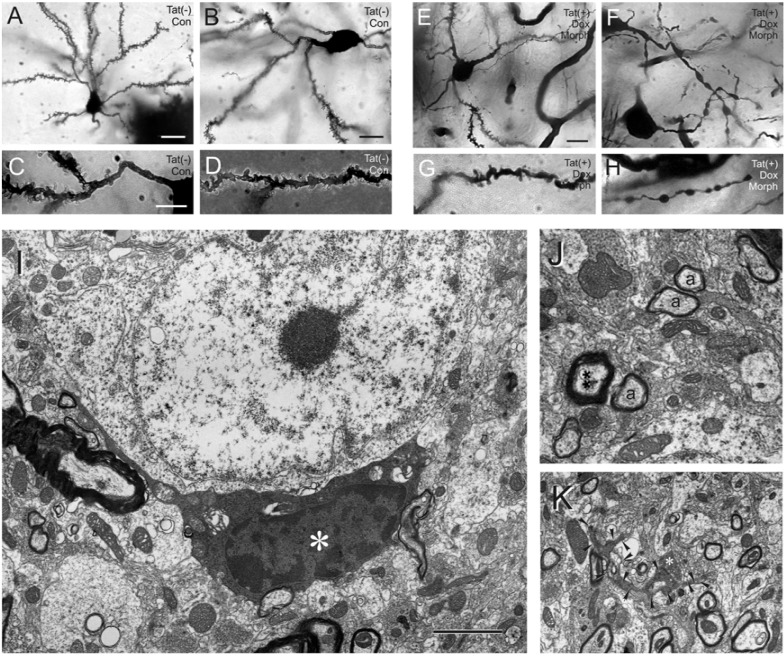
Golgi-impregnations of striatal neurons and dendrites from control Tat(-) (**A**-**D**) and Tat(+) (**E**-**H**) transgenic mice at 7 days following combined morphine exposure and Tat induction. The medium spiny neurons from Tat(-) mice are morphologically normal (**A**, **B**), and possess normal complements of spines on proximal (**C**) and distal (**D**) dendritic segments. By contrast, combined morphine exposure and Tat induction caused severe deficits in spine numbers and synaptodendritic injury (**E**-**H**), including severe dendritic varicosities and degeneration (**F**-**H**), that was worse than with morphine exposure or Tat induction alone (see reference [82] for detailed explanation); scale bar in **A** = 20 µm; the scale bars in **B**, **C**, **E** = 10 µm; **C**, **D**, **F**, **G**, **H** are the same magnification (note, figures E-H above correspond to figures R-U in ref [124]). Electron micrographs showing an abnormal oligodendrocyte (**I**) and myelin (**J**), and a degenerating dendrite in the striata of HIV-1 transgenic mice following 7 days of continuous Tat induction and co-exposure to morphine (**I**-**K**). An oligodendrocyte with abnormally dense cytoplasm with excessive vacuoles and condensed nucleoplasm (*) in close association with several myelinated axons (**I**); scale bar = 2 µm. Axons (**) that appear to be hypermyelinated compared to nearby axons of similar diameter (**a**) are quite common in the striata of Tat transgenic mice (**J**). Normally, myelin thickness is directly proportional to the diameter of the axon and is constant for axons of a particular diameter (**J**); the relationship is referred to as the g-ratio [306]. An electron dense dendrite (arrowheads) with a single presynaptic contact (*) appears to be degenerating (**K**); in all the above instances (**E**-**K**) morphine was continuously administered *via* a time-release pelleted implant (5 mg per day) (see [82]). (**A**-**H**): Reprinted from reference [124]; Copyright (2010), with permission from Elsevier and the American Society for Investigative Pathology.

In cerebral cortical neurons, morphine decreases NeuroD phosphorylation, which increases CaMKII phosphorylation and maintains excitatory dendritic spines [170]. CaMKII is an important target in neuroAIDS [185] and potentially pivotal in convergent opiate drug and HIV-1 interactions. The loss of spines impedes glutamate signals through α-Amino-3-hydroxy-5-methylisoxazole-4-propionic acid (AMPA) receptor (AMPAR) and NMDAR subtypes [102-104]. Excitotoxic injury reportedly originates from “extrasynaptic” NMDARs or *via *deleterious NMDAR subunit conformations [186, 187]. Alternatively, “synaptic” NMDAR signaling at excitatory PSD-95^+^ dendritic spines is reportedly neuroprotective [188, 188-190, 190, 191], and associated with specific subunit configurations, including NR3A [192] and NR2A [186] (through activation of phosphoinositide 3-kinase (PI3K)/Akt (protein kinase B or PKB), extracellular signal-regulated kinase (ERK), glycogen synthase kinase 3β, and/or FOXO [193-197]). Assuming chronic morphine exposure affects striatal neurons similarly, reductions in "favorable" spines are likely to be detrimental especially when confronted by an excitotoxic Tat or gp120 challenge (Fig. **1**). This may reveal a potential mechanism by which opiate drugs would aggravate the negative consequences of HIV-1 in medium spiny neurons.

Besides direct actions on the medium spiny neurons themselves, opioids can act by synergistically disrupting the function of MOR-expressing astroglia [70-72] and/or microglia [61, 80, 81]. In addition, morphine can excite dopamine neurons projecting from the ventral tegmental area (VTA) to striatal spiny neurons by hyperpolarizing inhibitory γ-aminobutyric acid (GABA)- expressing interneurons in the VTA [184]. Dopaminergic afferents into striatum from substantia nigra and VTA are markedly altered in neuroAIDS [65, 198-205], and are important targets for substance abuse [65, 200, 201, 206, 207]. Following chronic morphine exposure, GABA transporter function is disrupted leading to hyperexcitability of GABAergic neurons upon precipitated morphine withdrawal [208]. Assuming aspects of these findings can be generalized to striatal function, this would suggest that chronic opiate exposure causes sufficient maladaptive changes in both GABAergic and glutamatergic responsiveness (both presynaptically [209] and postsynaptically) to result in maladaptive neuron injury when confronted with HIV-1 Tat or gp120 [115, 124-126, 210].

Gp120 derived from CCR5 (R5)- or CXCR4 (X4)- tropic HIV-1 strains preferentially use CCR5 or CXCR4, respectively, as co-receptors for viral entry; dual-tropic strains can use either receptor. All strains are neurotoxic, but activate their receptors in specific ways that can induce effects similar to and/or different from those of the natural ligand [117]. This biased agonism can change the downstream signaling from beneficial or neutral to neurotoxic. For example, in the study cited above gp120 binding to CCR5 produces a neurotoxic response through activation of p38 MAPK even though CCR5’s natural β-chemokine ligands are neuroprotective. In comparison, CXCR4 activation by either gp120 or stromal cell-derived factor-1 (SDF-1 or CXCL12) leads to neuronal death. Others have reported that at a lower chemokine concentration the effects of SDF-1 and X4-tropic gp120 diverge, with SDF-1 phosphorylating both pro-survival (p-Akt) and pro-apoptotic c-Jun-terminal kinase (p-JNK) signals, whereas gp120 selectively activates pro-apoptotic signaling [211]. Strain differences have also been revealed in the production of brain-derived neurotrophic factor, which is suppressed by X4-tropic gp120 and enhanced by R5-tropic gp120 [212].

We found fundamental differences in the interaction of morphine with X4- compared to R5-tropic strains of HIV-1 in an infectious model of hepatitis C virus [213]. Prompted by these findings, we questioned whether morphine would interact with HIV-1 gp120 to cause neurotoxicity in a strain-dependent manner [60]. Interestingly, we found that morphine had no interactive cytotoxic effects with R5-tropic gp120_ADA_, while morphine caused a highly reproducible transient and coordinated acceleration of X4-tropic gp120_IIIB_ neurotoxicity. By contrast, there was a sustained additive neurotoxicity with combined exposure to morphine and bitropic gp120_MN_ [60]. While it is tempting to conclude that the coincident activation of both X4 and R5 co-receptors by gp120 imparts greater toxicity, it is equally reasonable to assume that gp120 displays agonist selective or biased agonism properties and that gp120 from each HIV-1 variant has a somewhat different effect. Lastly, although additional study is needed, in instances where morphine and gp120 co-exposure fails to show toxic interactions, a sound assumption may be that they are acting through a similar mechanism. This seems especially plausible considering the potential for interactions between MOR and CXCR4 or CCR5.

How opioids might directly (or indirectly through actions on glia) worsen the neurotoxic effects of gp120? One possibility is through overlapping actions at K^+^ channels. CXCR4-driven Gαq increases outwardly directed K^+^ currents [214]. Since chronic opioid exposure can lead to a loss in MOR coupling to inwardly directed K^+^ currents in neurons [16], and through mechanisms that may be partially dependent on glia [16], an exaggerated bias toward the outward movement of K^+^ may partially explain the interactive neurotoxicity seen with chronic opioid and gp120_MN_ co-exposure [60]. Gp120 appears to induce apoptosis in most neuron types studied through a caspase-3-dependent process [109, 115, 214-219]. How outwardly directed K^+^ activates caspase-3 is less certain [214], but is likely to contribute to gp120-induced imbalances in CXCR4 or CCR5 signaling in neurons through reduced ERK activation [211, 220-222] with accompanying overactivation of p38 [69, 108, 117, 223], JNK [108, 220, 224], and/or other MAPKs. More recently, Haughey *et al. *[225] described gp120-induced clustering of NMDARs in the dendrites of hippocampal neurons and subsequent declines in the excitotoxic threshold through facilitated increases in [Ca^2+^]_i_. The effects of gp120 on membrane clustering were partially blocked by the irreversible antagonist of CXCR4, plerixafor hydrochloride or AMD3100, indicating the involvement of CXCR4 signaling in NMDAR clustering [225]. Considering the known interaction(s) between MOR and CXCR4 [226-228], as well as maladaptive DOR-CXCR4 interactions unmasked in MOR knockout glia [229], the convergent effects represent a potential site where opiate drugs and gp120 interface in neuroAIDS [60, 230-232].

Meucci and co-workers propose that the ferritin heavy chain subunit can influence opiate drug toxicity by negatively regulating CXCR4 signals *via *Akt (PKB) [231, 232]. They go on to offer that otherwise neuroprotective CXCL12/SDF-1-CXCR4 homeostatic mechanisms normally present can be disrupted by increased ferritin heavy chain subunit expression [231]. This has considerable implications for X4-tropic gp120 and morphine neurotoxic interactions [60], implying that the degree of neurotoxicity may be “tunable” or modifiable by environmental or metabolic factors affecting the expression or trafficking of the ferritin heavy chain subunit. In addition, this may also be of considerable importance for opiate and X4-tropic gp120 interactive toxicity in oligodendroglia (Zou and Knapp, unpublished), since oligodendroglia are exquisitely dependent on ferritin heavy chain subunit expression for iron regulation, myelination, and their survival [233-235].

## OPIOID DRUGS EXACERBATE HIV-1-INDUCED NEURON DEATH THROUGH ACTIONS IN MOR-EXPRESSING GLIA

**Fig. (2):**
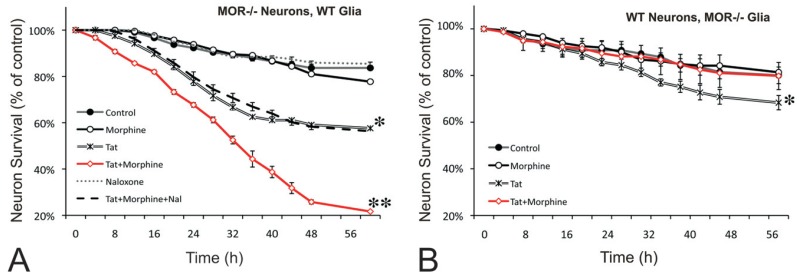
Morphine and HIV-1 Tat-induced interactive neurotoxicity is mediated by MOR-expressing glia. Synergistic HIV-1 Tat and morphine neurotoxicity was only evident when MOR-deficient striatal neurons were co-cultured with wild-type glia (**P < 0.01 versus all other groups, red line) (**A**), but not when wild-type neurons were co-cultured with MOR-deficient glia (**B**, red line). Co-cultures consisted of neurons and glia (~10:1 ratio of astroglia:microglia) derived from the striata of wild-type or MOR knockout mice. Note that HIV-1 Tat alone was neurotoxic to striatal neurons irrespective of MOR genotype (*P < 0.05 vs untreated controls, double lines) (see [95] for further explanation). Reprinted with permission from reference [95]; Copyright 2011 Oxford University Press.

In considering the convergence of HIV-1 and drug abuse, we proposed several years ago that critical interfacing of abused substances with HIV-1 occurs in glia [39]. More recent examination of opiate drug interactions with HIV-1 Tat protein provides further support for this concept and additionally suggests that the deleterious effects of opiates are principally mediated through direct actions at MOR-expressing astroglia and microglia (Fig. **2**) [95]. The near exclusive role of glia infers that the principal site(s) of opiate actions that exacerbate neuroAIDS occur directly on glia and not neurons. This crucial observation implies that key neurotoxic signals originate from opioid receptor-expressing glia. Lastly, despite the involvement of glia in interactive striatal neuron death [60, 95], it remains uncertain whether opiates and HIV-1 can directly affect more subtle forms of synaptodendritic injury. For example, morphine can directly affect spine reductions in cerebral cortical neurons [102, 103, 169, 236]; however, it is uncertain the extent to which morphine would similarly influence spine reductions in medium spiny neurons in the striatum or in pyramidal neurons within the CA1 region of the hippocampus. Defining the neural cell targets and aberrant glial-to-neuron signals (and vice versa) that mediate neuronal dysfunction and death are central toward understanding the pathogenesis of neuroAIDS in the context of opiate abuse.

**Fig. (3):**
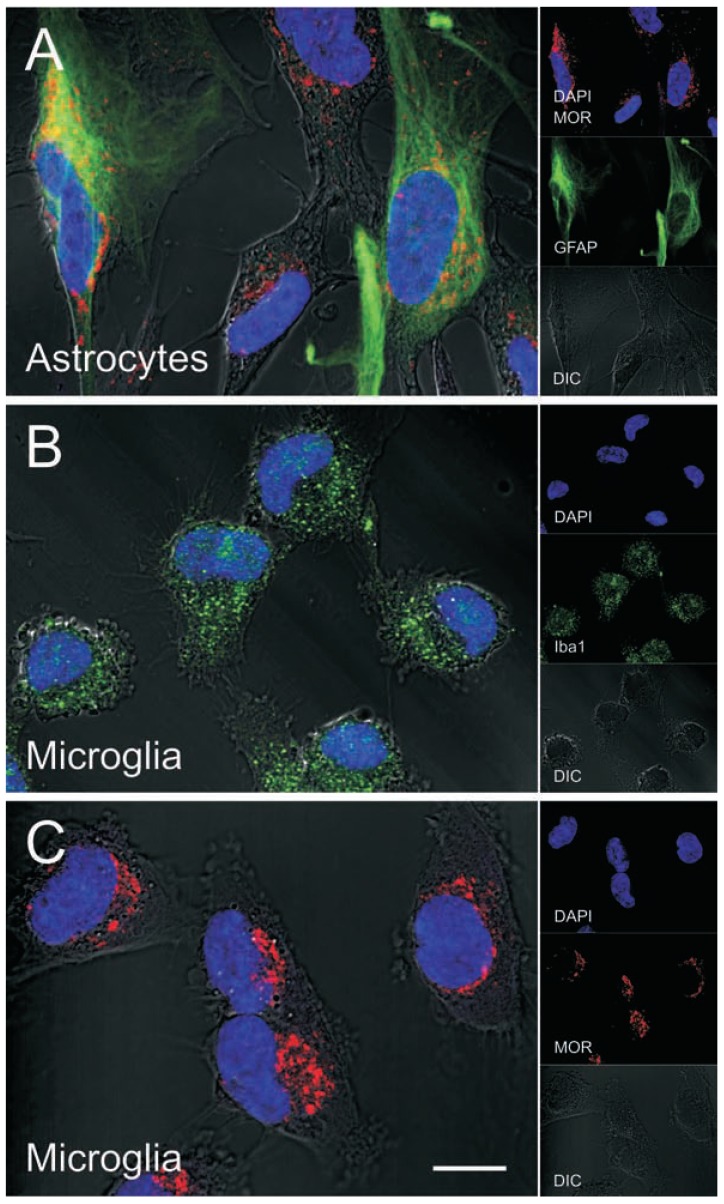
MOR immunofluorescence in human astrocytes and microglia. MOR and GFAP co-localization in subsets of primary human astrocytes (**A**). Astrocytes (catalog number 1800) were obtained from ScienCell Research Laboratories and cultured for 7-10 days according to the manufacturer's instructions. Iba-1 (**B**) and MOR (**C**) immunofluorescence in subpopulations of primary human microglia (ScienCell; catalog number 1900-f1) cultured as described for astrocytes. Cells were fixed with 3.7% paraformaldehyde, permeabilized with 0.5% Triton X-100, immuno-labeled, nuclei were stained with DAPI (blue), and images were enhanced by differential interference contrast (DIC) optics. Primary antibodies used were MOR (epitope within amino acids 1-15 of the N-terminus of human MOR) (Novus Biologicals, catalog number NBP1-31180), GFAP (Millipore, catalog number MAB360), and Iba-1 (Wako, catalog number 019-19741); all at a 1:200 dilution. Images were acquired using a Zeiss LSM 700 laser scanning confocal microscope at 63x (1.42 NA) magnification and ZEN 2010 software (Carl Zeiss Inc, Thornwood, NY), and edited using ZEN 2009 Light Edition (Zeiss) and Adobe Photoshop CS3 Extended 10.0 software (Adobe Systems, Inc.).

Opioid signaling is highly contextual and fundamentally different in each cell type [237-239]. For this reason, defining the distinct and cell specific targets that are engaged by opiates to trigger CNS inflammation and neuronal injury is critical toward understanding how opiate abuse worsens the disease. A contributing factor in differential opioid signaling may be the vast amount of alternative splicing of MOR [239], and the variant splicing differences in different cell types is a largely unexplored area [45]. Given that subpopulations of astroglia and microglia (Fig. **3**), as well as neurons can express MOR, future evaluation of the MOR variant expression profile in these individual cell types is of great importance. Furthermore, defining differential signaling cascades associated with each MOR variant, and determining whether HIV-1 affects expression or preferentially interacts with particular MOR variants in humans (Fig. **3**), may shed additional light on the intracellular pathways in glia that trigger glia-mediated neurotoxicity in HIV-1-infected opiate abusers.

## HOW MIGHT OPIATE-EXPOSED GLIA MEDIATE BYSTANDER TOXICITY IN NEURONS?

### Critical Importance of Neurotoxic Intercellular Signals

If, as noted above, HIV-1 is a glia-specific disease and opiate drug abuse imparts significant neurotoxicity through actions in MOR-expressing astroglia and microglia, then identifying the specific, bidirectional intercellular glia-neuron signaling events driving bystander neuron injury and death is of critical importance for understanding the pathogenesis. What is the nature of the intercellular signals that might be affected by opiates? Many glially derived signals that are likely to affect neuron injury or the maintenance of synapses have been identified [39, 60, 95, 96, 151, 240-243]. Several factors have been assumed to be involved in opiate drug toxicity because they are known to be modified by opiate abuse and because of their established importance in experimental models or clinical studies of HIV-1 neuropathology, but without direct testing for an interaction. Prime examples of these are excessive extracellular glutamate, extracellular ROS, and reactive nitrogen species, long proposed as "known suspects" in HIV-1 neuropathogenesis [115, 116, 244-247]. When we directly tested whether these “presumed” interactions were in fact operative in HIV-1 Tat and opiate-mediated neurotoxicity, all appear to be complicit to varying degrees, but none actually drive the interactive pathogenesis.

#### Glutamate.

Glutamate has long been thought to contribute to the generalized excitotoxicity attributed to HIV-1-induced neuron injury and/or death. These effects are not limited to a particular viral protein since HIV-1 gp120, Tat, and intact virions can all contribute to excess glutamate in the extracellular milieu and hyperexcitability in neurons. Gp120 and intact virions restrict glutamate uptake by astroglia through inhibition of excitatory amino acid transporter 2 (EAAT2) [183]. EAAT1 is altered by HIVE and may respond to cART-induced reductions in encephalitis [248, 249]. The activation of the Na^+^/H^+^ exchanger is another potential target for HIV-1 [250].

Exposure to either morphine or HIV-1 Tat by itself inhibits buffering of a glutamate challenge, and this occurs largely through effects on astrocytes [60, 95]. Although combined Tat and morphine tended to further reduce glutamate-buffering ability, the interaction was not significant [95]. Therefore, reduced glutamate buffering did not correlate with increases in neuron death due to morphine and Tat co-exposure. Gp120 and morphine similarly failed to block glutamate uptake in an additive manner, although either treatment by itself did prevent enriched astroglial cultures from responding appropriately to a glutamate challenge [60].

In all of the above studies, excess glutamate was applied to cultures enriched in astroglia (90.2 ± 0.4%) and microglia (8.8 ± 0.6%). Under normal physiologic conditions, EAAT1 and EAAT2 are predominantly expressed by astroglia and minimally expressed by microglia [251, 252]. However, specific proinflammatory triggers, including infection with SIV_mac251_ in non-human primates, can increase the expression of EAAT1 and EAAT2 in macrophages or microglia [253]. Interestingly, the particular inflammatory mediators involved can differ among species including humans and mice (reviewed in [251]), and effects in murine microglia may not be generalizable to humans. Since astroglia outnumber microglia about 10:1 in our striatal mixed-glial cultures [95], astroglia were assumed to be the principal cell type involved. Moreover, since there were no net increases in extracellular glutamate in the presence of the EAAT1-5 inhibitor, DL-threo-b-benzyloxyaspartate (DL-TBOA), or the EAAT1-3 inhibitor, (2S, 3S)-3-[3-[4 (trifluoromethyl) benzoylamino] benzyloxy] aspartate (TFB-TBOA), it is assumed that the inability of glia to deplete the glutamate increases caused by Tat ± morphine exposure was due to restricted uptake [95]. Despite our efforts to distinguish between reduced uptake and enhanced release, the assay may be relatively insensitive to transient and subtle alterations in glutamate release [254]. For example, under appropriate conditions, the activation of CXCR4 can cause glutamate release from astroglia [254]. Moreover, this assay is obviously unable to discriminate synaptically versus extrasynaptically directed glutamate release.

Regarding disrupted astroglial function, a compelling question regards the consequences of opiate abuse and HIV-1 on the function of the “tripartite synapse” and “gliotransmission”. Evidence is clear that astroglia affect synaptic transmission and plasticity [255-258]. Glutamate and D-serine released from astrocytes at the synapse are essential for synaptic plasticity [259], as defined by changes in long-term potentiation in hippocampal neurons (reviewed in [256]). By restricting EAATs that remove extracellular glutamate [60, 95], HIV-1 proteins and opiates are likely to affect gliotransmission indirectly, as well as permit excess glutamate to accumulate at extrasynaptic sites. Moreover, chronic morphine exposure significantly depresses EAAT2 expression in the striatum and hippocampus [260], and EAAT1 and EAAT2 expression in the spinal cord [261], while opiate withdrawal dramatically increases EAAT2 transcripts in the striatum [260].

While the net consequences of opiate drug and HIV-1-induced alterations in the response of astroglia to extracellular glutamate may be largely attributable to EAAT1 and EAAT2 function, the glial response may not be limited to altered EAAT function and appears to differ among glial types. By contrast to observations in cultures containing a large proportion of astrocytes, in cultures of isolated microglia, HIV-1 Tat exposure markedly increases glutamate secretion, while morphine co-exposure can significantly increase glutamate secretion above levels seen with Tat alone through actions involving the x_c_^-^ cystine-glutamate antiporter [80]. Lastly, as noted, EAAT1 and EAAT2 are minimally functional in resting microglia, but are inducible with immune activation [251]. The extent that more protracted exposure to opiates or HIV-1 proteins upregulates or fails to upregulate EAAT1 and EAAT2 expression in microglia is uncertain but a potentially important consideration.

#### Cytokines and chemokines.

The role of cytokines, and especially chemokines, in intercellular signaling from infected microglia to neurons in neuroAIDS is well established and has been extensively reviewed elsewhere [20, 115, 262-271]. Based on these and other findings within the CNS, we tested whether opioids might affect the release of pro-inflammatory cytokines and chemokines by astroglia and microglia exposed to HIV-1 proteins. The findings indicated that opioids could accelerate and enhance the release of cytokines and chemokines caused by HIV-1 Tat [71, 72, 124], as well as gp120 exposure in some instances [86, 181, 272]. Much of this data has been reviewed previously [13, 39].

Elevated levels of fractalkine/CX_3_CR1 have been found in the brains and CSF of patients with HIVE, and have been localized to both neurons and astroglia [273, 274]. These and other findings suggest that elevations in fractalkine by neurons facilitate interactions with CNS immune cells and it was demonstrated that moderate levels of exogenously administered fractalkine were able to protect against Tat or gp120 mediated neurotoxicity [222, 274, 275]. We recently reported that exogenous fractalkine/CX_3_CL1 can be neuroprotective against the deleterious effects of morphine and HIV-1 Tat co-exposure [96]. Collectively, the above findings suggest a potential therapeutic course for fractalkine in neuroAIDS. Although the cellular mechanisms underlying the observed neuroprotection are not certain, findings that exogenous fractalkine reduces microglial motility and fails to protect neurons co-cultured with *Cx3cr1*^-/-^ mixed glia suggest that fractalkine acts by interfering with toxic microglial-neuron interactions. In addition to its blockade of Tat and morphine mediated neurotoxicity, fractalkine and its receptor have more direct interactions with gp120 [222]. CX_3_CR1 acts as a co-receptor for viral entry [276], and thus fractalkine is able to displace gp120 on the fractalkine receptor to prevent microglial activation. Additionally, CX_3_CL1-CX_3_CR1 interactions can attenuate gp120-mediated neurotoxicity by preventing microglial activation and upregulation of pro-survival factors such as p-Akt [222].

#### Glial heterogeneity.

**Fig. (4):**
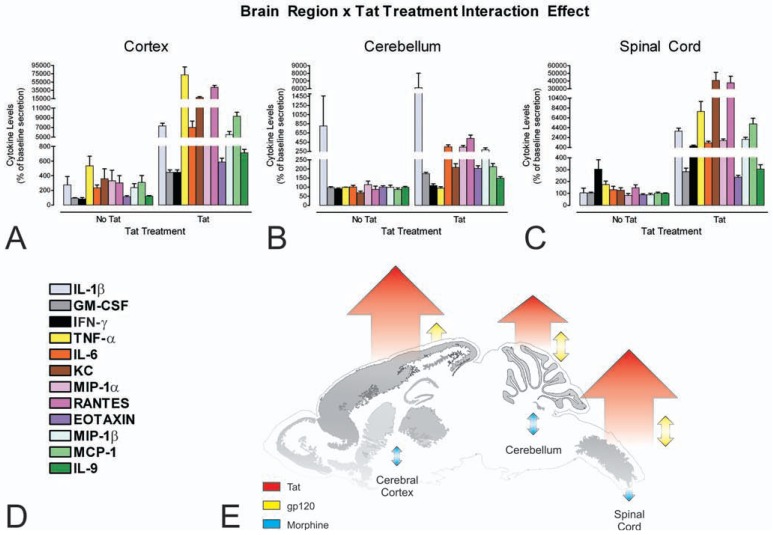
Astroglia isolated from the cerebral cortex (**A**), cerebellum (**B**), and spinal cord (**C**) display significant regional differences in the pattern of cytokine release in response to HIV-1 Tat *in vitro* (**A**-**C**). Interestingly, the pattern of cytokine release in response to Tat paralleled the incidence of HIV-1-related neuropathology in the brain and spinal cord (see reference [86]). Striatal astrocytes, analyzed as part of another study, show a far more dramatic interaction between HIV-1 Tat and morphine [72]. Cytokines and chemokines were analyzed simultaneously by multiplex suspension array assays [86]; legend provided in (**D**). Overall responses to HIV-1 Tat, gp120 and morphine across brain regions are summarized (**E**) (see text and reference [86] for further explanation). Reprinted with permission from reference [86]. Copyright 2010 American Chemical Society.

Astroglia and microglia are phenotypically diverse in terms of their expression of opioid system peptides and receptors. Subsets of astrocytes can express MOR, DOR, and/or KOR [73, 75, 76, 81, 277, 278], as well as the proenkephalin opioid gene and proenkephalin-derived peptides, including Met-enkephalin, Leu-enkephalin, and partially processed enkephalin precursors [79, 279-284]. The heterogeneity within astroglia is not limited to the opioid system, as other neurochemical systems similarly show a high degree of phenotypic diversity [285-289], a degree that is perhaps only rivaled by neurons. Glial heterogeneity exists even within a single brain region. For example, astroglia in the striatum vary individually in their expression of MOR, DOR, and KOR [73]. Heterogeneity in the expression of endothelin-1, and α1-adrenergic and muscarinic [290] receptors is present among astroglia in other brain regions. Astroglia also display significant regional differences in their functional response to HIV-1 Tat and gp120 (Fig. **4**).

**Fig. (5):**
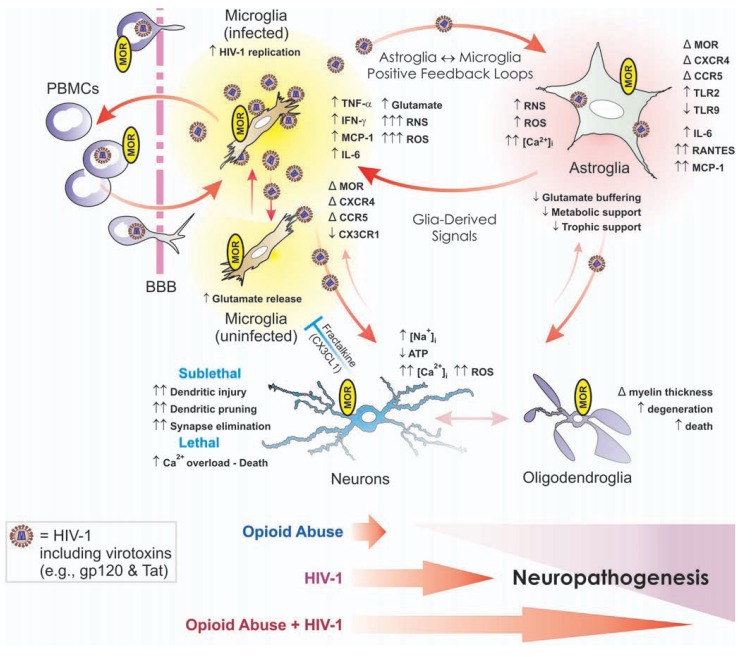
Opiate drugs exacerbate HIV-1 neuropathogenesis through direct actions on glia, especially microglia and astroglia, in addition to affecting neurons. In the CNS, HIV-1 infects microglia, and to a lesser extent astroglia, causing the production of reactive oxygen and nitrogen species (ROS and RNS, respectively), pro-inflammatory cytokines, and the release of HIV-1 proteins such as gp120 and Tat, which promote inflammation and cytotoxicity in bystander neurons and glia. Chronic opiate abuse by itself can cause some neuropathology (e.g., see [93]); however, in HIV-1-infected individuals opiates can potentiate many of the pathophysiological effects of the disease—especially in the central nervous system. Multiple neuronal and glial types can express the µ-opioid receptor (MOR). Many of the neurodegenerative effects of opioid-driven synergy arise through direct actions on microglia and astroglia. In fact, evidence suggests that reverberating inflammatory/cytotoxic positive feedback signaling between HIV-1-infected microglia and astroglia is exacerbated by opiate exposure—revealing novel targets for therapeutic intervention in opioid drug abuse and HIV-1 comorbidity. Abbreviations: α-chemokine “C-X-C” receptor 4 (CXCR4); altered or changed (Δ); β-chemokine “C-C” receptor 5 (CCR5); blood-brain barrier (BBB); decreased (↓); fractalkine (CX3CL1); fractalkine receptor (CX3CR1); increased (↑); interferon-γ (IFN-γ); interleukin-6 (IL-6); intracellular Ca^2+^ concentration ([Ca^2+^]_i_); intracellular sodium concentration ([Na^+^]_i_); monocyte chemoattractant protein-1 (MCP-1 [or CCL2]); peripheral blood mononuclear cells (PBMCs); regulated upon activation, normal T-cell expressed, and secreted (RANTES [or CCL5]); Toll-like receptor (TLR). Fractalkine released by neurons (and astroglia) can be neuroprotective by limiting the neurotoxic actions of microglia (blue “┴”); red arrows suggest pro-inflammatory/cytotoxic interactions. Modified and reprinted from reference [307], Copyright (2006), with permission from Springer.

Unlike neurons, astroglial characteristics appear modifiable by adjacent neurons and perhaps other regional and extrinsic cues within the extracellular milieu [287, 291, 292] (Fig. **5**). For instance, intrastriatal injection of Tat significantly increases the number of MOR immunoreactive astroglia at 48 h post-injection [72], while the proportion of MOR immunoreactive neurons remains unchanged (Hauser, unpublished). The diversity and plasticity of receptor expression by astrocytes is not limited to neurotransmitter receptors. Pattern-recognition receptors (PRRs), which recognize conserved microbial molecular motifs and are critical components of the innate immune system, show considerable diversity in astroglia. PRRs expressed by astrocytes include multiple members of the Toll-like receptor (TLR) family including TLR2, TLR3, TLR4, and TLR9 [106, 293]. Importantly, exposure to HIV-1 and/or opiates can alter the expression of TLR2 and TLR9, which are important in the host defense response to HIV-1. Chronic opiate exposure may predispose the CNS to HIV-1 infection by suppressing the innate immune response and by inhibiting TLR9 expression by astrocytes [293]. Morphine suppresses the response of alveolar macrophages against *Streptococcus pneumoniae* pathogenicity by altering TLR9-dependent NF-κB signaling [294]. Morphine reportedly can induce apoptosis in neurons *via *TLR2 [295]. Recently, morphine has been proposed to increase nociception and inflammation through a non-canonical mechanism involving direct interactions with TLR4 in glia [296, 297], although the detailed molecular mechanisms underlying the interactions remain to be elucidated. Collectively, the above results suggest that opiate drugs can affect the innate immune response through MOR-dependent alterations in one or more TLR signaling pathways and perhaps *via *novel MOR-independent actions at TLR4.

#### Oligodendroglia and demyelination.

Oligodendroglia within the striatum are highly sensitive to the effects of morphine in HIV-1 Tat transgenic mice [83]. Striatal oligodendrocytes are the only cell type in the Tat transgenic mice to die, albeit at very low (~1.5%) rates, within 2 to 7 d of combined morphine exposure and Tat induction [83] (Fig. **1**). Besides cell death *per se*, there is considerable evidence of cellular degeneration in Golgi silver-impregnated oligodendroglia, and a reduction in the number and extent of myelinating processes in a subset of cells [83]. The effects of Tat and morphine on oligodendrocytes are likely to be direct. Unlike astrocytes, oligodendrocytes can express NMDARs [298-300], a molecular target of HIV-1 Tat in neurons [126, 130, 137]. Furthermore, oligodendrocytes can express MOR and KOR [277, 279, 301], and immature oligodendrocytes appear to be particularly sensitive to the effects of MOR and KOR activation [302-305]. Perinatal exposure to buprenorphine, a partial MOR agonist and partial KOR antagonist, results in aberrant myelin g-ratios [303], indicating an inappropriate relationship between myelin thickness:axon diameter and inferring that intercellular neuron-to-oligodendroglia signaling is disrupted [306]. Taken together, the above findings suggest that oligodendrocytes are preferentially vulnerable to HIV-1 and opiate drug interactions [83] and this may be an additional mechanism by which opiate-HIV-1 interactions in glia result in neuronal dysfunction and injury (Fig. **5**).
